# Supplementary material for: Mechanical Performance of 3D-Printed Polyethylene Fibers and Their Durability against Degradation
Source: Materials (Basel). 2023 Jul 24;16(14):5182. doi: 10.3390/ma16145182 (PMC10386389; doi:10.3390/ma16145182)
Supplement: Supplementary file 1 [file materials-16-05182-s001.zip › materials-2447795-supplementary.pdf]

materials

Article

Mechanical Performances of 3D-Printed Polyethylene Fibers and Their Durability Against Degradation

Yao Xiao [1,2,+], Shikai Zhang [2,+], Jingyi Chen [2,3], Baoling Guo [1,3*] and Dong Chen [2,3,4*]

[1] Department of Oncology, Longyan First Affiliated Hospital of Fujian Medical University, Longyan 364000, China; 11927061@zju.edu.cn
[2] College of Energy Engineering and State Key Laboratory of Clean Energy Utilization, Zhejiang University, Hang-zhou 310003, China; skzhang@zju.edu.cn
[3] John A. Paulson School of Engineering and Applied Sciences, Harvard University, Cambridge, MA 02138, USA; jchen1@g.harvard.edu
[4] Zhejiang Key Laboratory of Smart Biomaterials, College of Chemical and Biological Engineering, Zhejiang University, Hangzhou 310027, China; chen_dong@zju.edu.cn
+These authors contribute equally to this work.
* Correspondence: baolingguo@sina.com; chen_dong@zju.edu.cn

Supplementary Materials

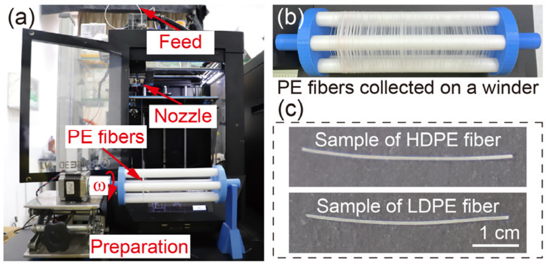

Figure S1. Preparation of PE fibers. (a) Experimental setup for the preparation of PE fibers. (b) PE fibers collected on a winder. (c) Samples of HDPE and LDPE fibers cut into 5 cm long.

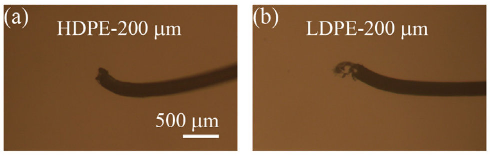

Figure S2. Fracture surface morphology of 3D-printed PE fibers after tensile fracture. (a) HDPE-200 μm. (b) LDPE-200 μm. The printer head temperature is 215 °C.

Table S1. Mechanical properties of HDPE and LDPE fibers with different diameters. The printer head temperature is 215 °C. HDPE and LDPE fibers with a diameter of 200 μm, 400 μm and 600 μm are prepared with a collecting speed of 11.6 cm/s, 3.1 cm/s and 1.2 cm/s, respectively. If not specified, Young's modulus is determined at 1% strain.

Sample	Tensile Strength (MPa)	Tensile Strain (%)	Young's Modulus (GPa)	Toughness (MJ/m³)
HDPE-200 μm	50.6±1.4	426±30	0.976±0.073	171±20
HDPE-400 μm	22.4±3.7	809±93	0.867±0.042	178±32
HDPE-600 μm	18.7±1.3	917±62	0.639±0.088	172±17
LDPE-200 μm	31.6±1.4	824±63	0.364±0.096	195±28
LDPE-400 μm	14.8±1.3	1335±156	0.093±0.038	184±32
LDPE-600 μm	8.51±0.87	1073±100	0.071±0.005	89.0±7.6

Table S2. Mechanical properties of HDPE and LDPE fibers printed at different printer head temperatures. The diameter of HDPE and LDPE fibers is about 200 μm.

Sample	Tensile Strength (MPa)	Tensile Strain (%)	Young's Modulus (GPa)	Toughness (MJ/m^3)
HDPE- printed at 215 °C	50.6±1.4	426±30	0.976±0.073	171±20
HDPE- printed at 235 °C	40.2±3.7	543±50	0.760±0.042	208±30
HDPE- printed at 255 °C	36.4±4.0	570±89	0.628±0.101	213±48
LDPE- printed at 215 °C	31.6±1.4	824±63	0.364±0.096	195±28
LDPE- printed at 235 °C	26.9±2.4	952±63	0.259±0.031	200±25
LDPE- printed at 255 °C	25.3±1.8	997±99	0.224±0.008	202±30

Table S3. Mechanical properties of HDPE and LDPE fibers before and after UV exposure. The printer head temperature is 215 °C. The diameter of HDPE and LDPE fibers is about 200 μm. The samples are placed 8 cm under a UV lamp (395 nm, 18 W).

Sample	Tensile Strength (MPa)	Tensile Strain (%)	Young's Modulus (GPa)	Toughness (MJ/m^3)
HDPE-0 h UV	50.6±1.4	426±30	0.976±0.073	171±20
HDPE-12 h UV	46.6±3.4	380±42	0.968±0.095	151±30
HDPE-24 h UV	35.2±6.0	332±83	0.691±0.048	111±36
HDPE-48 h UV	33.9±3.8	324±29	0.630±0.148	113±21
LDPE-0 h UV	31.6±1.4	824±63	0.364±0.096	195±28
LDPE-12 h UV	30.3±1.8	807±32	0.283±0.008	180±7
LDPE-24 h UV	20.8±2.1	621±93	0.041±0.012	101±26
LDPE-48 h UV	17.3±4.1	531±90	0.030±0.012	75.5±24.3

Table S4. Mechanical properties of HDPE and LDPE fibers before and after thermal degradation. The printer head temperature is 215 °C. The diameter of HDPE and LDPE fibers is about 200 μm. HDPE and LDPE samples are baked in an oven of 100 °C.

Sample	Tensile Strength (MPa)	Tensile Strain (%)	Young's Modulus (GPa)	Toughness (MJ/m³)
HDPE-0 h 100°C	50.6±1.4	426±30	0.976±0.073	171±20
HDPE-12 h 100°C	47.0±5.5	363±73	0.751±0.161	147±35
HDPE-24 h 100°C	43.5±4.0	289±26	0.704±0.132	125±36
LDPE-0 h 100°C	31.6±1.4	824±63	0.364±0.096	195±28
LDPE-12 h 100°C	19.8±4.3	615±94	0.122±0.009	113±29
LDPE-24 h 100°C	18.6±1.0	386±33	0.102±0.067	62.3±5.7

Disclaimer/Publisher's Note: The statements, opinions and data contained in all publications are solely those of the individual author(s) and contributor(s) and not of MDPI and/or the editor(s). MDPI and/or the editor(s) disclaim responsibility for any injury to people or property resulting from any ideas, methods, instructions or products referred to in the content.
